# Educational

**Published:** 1895-07

**Authors:** 


### EDUCATIONAL.

Dr. J. H. Wattles, of Kansas City, Missouri, has been investigating the sources of supply of the milk of that city, and has aroused a keen interest in this all-important question among the health officers and city guardians. The commercial aspect of the milk-supply as to any adulterations or robbing of cream has been for some time under the surveillance of the city Board of Health, but the condition of the animals and their environments from whence the milk is obtained did not occur to them as of much importance until awakened by the investigations of Dr. Wattles.

Kansas City is far from being alone in this happy easy-of-mind state of affairs. Almost every large city in the country needs a rude awakening. The city has been asked to appoint a milk and dairy inspector, whose province it will be to examine into the condition of the places of production, the animals themselves, and allow the sale of no milk that does not bear with it a certificate of satisfactory inspection. Dr. Wattles well says to the people and officers of the law of Kansas City that such an inspector should be a well-equipped veterinarian, as veterinarians are the only ones qualified to perform this work properly.

